# Uncovering the hidden mechanics of upper body rotations in tennis serves using wearable sensors on Dutch professional players

**DOI:** 10.3389/fspor.2024.1463299

**Published:** 2025-01-07

**Authors:** B. van Trigt, E. Faneker, A. J. R. Leenen, A. E. Hoekstra, M. J. M. Hoozemans

**Affiliations:** ^1^ Department of Biomechanical Engineering, Delft University of Technology, Delft, Netherlands; ^2^ Ridgeline Movement, Amsterdam, Netherlands; ^3^ Department of Human Movement Sciences, Faculty of Behavioural and Movement Sciences, Vrije Universiteit Amsterdam, Amsterdam, Netherlands; ^4^ Thirty Love Academy, Diemen, Netherlands; ^5^ Royal Netherlands Lawn Tennis Association (KNLTB), Amstelveen, Netherlands

**Keywords:** intersegmental timing, ball speed, biomechanics, kinematics, angular velocity, kinetic chain, IMU system

## Abstract

**Background:**

It is assumed that the tennis serve is performed according to the kinetic chain principle in which a proximal-to-distal sequence in peak angular velocities of subsequent body segments can be observed to reach high end point ball velocities. The aim of the present study was to investigate if the magnitude and (intersegmental) timing of peak angular velocities of body segments in professional tennis players are different between first and second serves and if they are associated with serve performance.

**Methods:**

Eight (two female and six male) professional tennis players performed each 48 tennis serves on a tennis court. Serve performances: Ball speed and accuracy were measured with a PlaySight system. Kinematics were assessed with a custom made high-end inertial measurement units (IMUs) system, sampled at 1,000 Hz. Magnitudes of, as well as the intersegmental timing between, three dimensional peak angular velocities of the pelvis, trunk, and dominant upper arm were analysed in relation to ball speed and accuracy with generalized estimating equations.

**Results:**

Peak angular velocities of the pelvis, trunk and upper arm were significantly higher in the first compared to the second serve. The intersegmental timing did not show significant differences. Also, the intersegmental timing was not associated with the ball speed. Ball speed was significantly positively associated with peak angular velocities of the trunk and upper arm on both the first and second serve. Accuracy was positively associated with the peak trunk angular velocity and intersegmental timing between the pelvis and trunk in the first serve. Accuracy was negatively associated with peak trunk angular velocity in the second serve.

**Conclusion:**

The arm movement is important to produce high ball speed during a tennis serve. Additionally, the trunk, proximal to the upper arm in the kinetic chain, showed associations with ball speed. In contrast to the upper arm also with accuracy. Interestingly, professional players do not strictly follow a proximal-to-distal sequence. Intersegmental timing appears to be less important in the tennis serve compared to the segmental angular velocities, which were higher in the first compared to the second serve. Future research should investigate the uncovered role of the trunk in relation to tennis serve performance.

## Introduction

The tennis serve is often regarded as the most crucial and complex stroke in competitive tennis. It is used to start each point or try to end each point immediately. To succeed this, the combination of the highest ball speed and ball spin is important while maintaining a sufficient accuracy level (1). In professional tennis, ball speed surpassing 200 kph when serving are reached frequently. A complex coordinated whole-body action is required to generate and transfer kinetic energy from the lower extremity up to the upper extremity to ultimately end in high ball speeds (2, 3). The term kinetic chain is often used to describe this coordinated whole-body movement that occurs in a proximal-to-distal sequence (3, 4). This sequence can be explained by summation of speed principle that states that the distal segment start accelerating their rotational motion when the adjacent predecessor reaches its maximum rotation. The aim is to maximize the highest possible velocity of the last segment in the chain (5).

The kinetic chain for the tennis serve is initiated at the feet, progressing to the pelvis, trunk, upper arm, forearm, and finally to the hand and racket (6). Various studies have identified upper-limb proximal-to-distal segment sequencing in the tennis serve (7, 8). These studies found significant and positive correlations between segmental angular velocities and ball speed. The timing of rotations between the segments, i.e., intersegmental timing, which is an important aspect of the mechanics of the kinetic chain, were not included in these studies. In other overhead sports, like baseball pitching, it is shown that the relative time intervals (or intersegmental timing) between peak segment angular velocities are associated with end-point ball speed (9, 10). Thus, the combination of intersegmental timing with angular velocities seems important with ball speed. In the tennis serve, Martin et al. (11) showed that the transmission between mechanical energy from the trunk to the hand was positively associated with ball speed. Therefore, besides the magnitude of segmental angular velocities it is important to consider the effect of intersegmental timing in relation with serve performance.

Segmental angular velocities and intersegmental timing can be accurately calculated in the laboratory with motion capture systems (10). However, measurements in the laboratory contain several drawbacks as they are time-consuming, require financial and human resources, and can thus often include only single-time assessments. A recent study in baseball pitching showed that it is possible to measure the trunk and pelvis angular velocity with an inertial measurement unit (IMU) sensor system (12). This sensor system is highly practical tò use and offers many possibilities for coaches and players in the future, as it can be used anywhere and anytime.

In tennis, most of the time, the first serve is a flat serve resulting in higher ball speeds and a lower serve percentage of in/out. Accuracy becomes more important in the second serve, because if the ball is hit outside the serve box the player loses the point immediately (13). Therefore, higher serve percentages in the second serve are shown which is possibly a result of more safely hit kick or slice serves (13). Understanding whether there are differences in the kinetic chain between first and second serves in relation to serve performance will help formulate training plans and understand how the body behaves.

Typically, a tennis player's serve performance can be assessed quantitatively by counting the number of aces and serve winners or calculating the percentage of points won when serving. However, quantifying serve performance in this way is largely dependent on the opponent's return of serve. Well-documented, more isolated serve performance indicators are the parameters ball speed and ball bounce accuracy (14). These indicators combined discriminate most reliably between performance levels (15). In relation to serve performance, analysis of the timing between body segment rotations, as well as the magnitudes of peak angular velocities of the body segments themselves, can provide a better understanding of the relation between the kinetic chain in the tennis serve and serve performance.

The first aim of this study is to investigate if there is a difference in peak angular velocities of upper body segments and intersegmental timing between the first and second serve in professional tennis players. The second aim is to investigate if the tennis serve performance is associated with magnitudes of peak angular velocities of body segments and the intersegmental timing between these peak angular velocities.

## Methods

### Participants

Eight participants (2 female, 6 male) participated in the study (mean age 21.5 SD 2.1 years, body height 183.4 SD 5.7 cm, body mass 76.7 SD 11.9 kg). All players were fulltime, professional players training with the Dutch tennis federation (Royal Dutch lawn Tennis Association, KNLTB). All players had a WTA or ATP ranking except for one, who had a junior's ITF ranking. The ATP/WTA ranking of the participants in increasing order at the time of measuring was: 20 (junior ITF), 173, 345, 489, 598, 696, 745, 1,082. Informed consent forms were distributed and signed prior to data collection. No player reported to suffer from severe injury or had undergone recent surgery (in the last three months) in the dominant upper extremity. The study protocol was performed in accordance with the Code of Ethics for the Social and Behavioural Sciences (4) and had been approved by the Science and Ethics Committee (VCWE) under registration number VCWE-2020-022.

### Procedure

**Figure 1:**
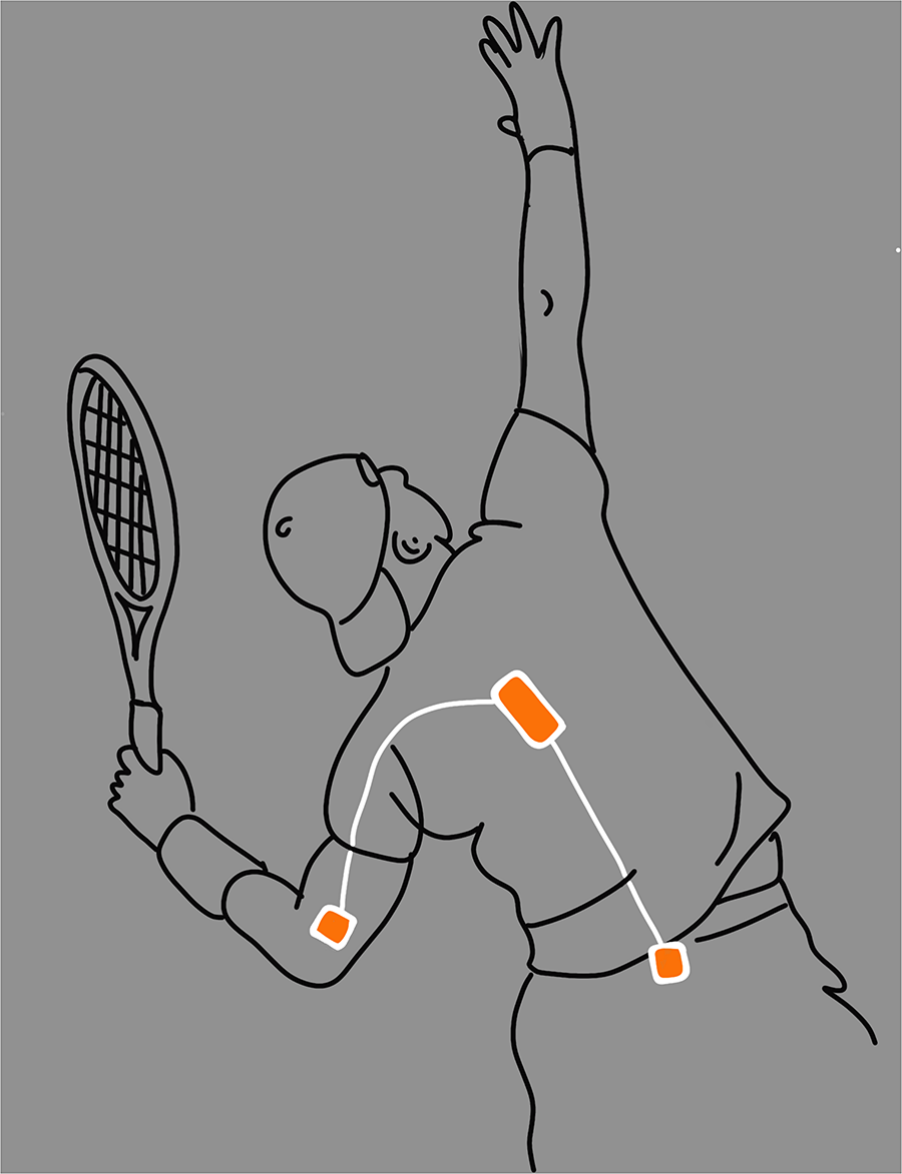
Shows the attachment of the IMU-system on the participant. All sensors were placed on the skin under the clothes. The pelvis sensor was attached right above the sacrum on the spine, at the centre of the line between the spinae iliaca posterior superior. The trunk sensor was placed at the point of the fifth thoracic vertebra. The upper arm sensor was attached at the distal point of the humerus, posterior midpoint between the lateral and medial epicondyle.

Data collection was performed at the indoor National Training Centre of the KNLTB. Three wired inertial measurement units (IMUs) were placed on the pelvis, trunk and dominant upper arm (Figure 1). The IMUs were attached with double sided tape on the skin and covered with medical tape. Participants performed individually several serves for three minutes to warm-up and get used to sensors placed on the body. After warming-up, participants were instructed to hit 48 serves in total with a break of 90 s after 24 serves. Participants were asked to hit the serves as they would do in an official singles match. The serves were hit in pairs, that is, a first serve followed by a second serve. The research leader called out the serve location together with the required aiming target in sets of two, e.g.: “Deuce side: first serve T, second serve wide”. This was repeated 12 times before the break, and 12 times again after the break. The sequence of aiming targets (either T, body or wide) was randomized and equal for all players. The protocol of the test can be found in more detail in the Appendix. When the ball hit the net but still bounced in the correct service box (a “let”), the participant was instructed to produce the same serve again (either first or second serve with the same target), in line with the official tennis rules that state that a service let can be replayed.

### Data acquisition

Three-dimensional body kinematics were assessed with a custom-made IMU system (Figure 1). The triaxial gyroscopes of the IMU system were used to measure the segmental angular velocities. A custom-made IMUs system was used because the arm movement during a tennis serve exceed the range (±2,000 deg/s) of commercial available gyroscopes of the IMUs. The pelvis and thorax IMU contained an integrated triaxial accelerometer of ±30 G and a triaxial gyroscope of ±4,000 deg/s, while the upper arm IMU contained an integrated triaxial accelerometer of ±200 G and a triaxial gyroscope of ±20.000 deg/sec. The triaxial gyroscopes were used to measure the angular velocity of the segments. The IMUs of the system had a neglectable mass (<5 g) and were connected with wires to have an optimal time-synchronization. The collected data was sampled at 1,000 Hz.

Ball speed and ball bounce location were measured using a PlaySight Smartcourt Pro System (PlaySight, 5.3.301.0, New York, USA) with use of ten on court cameras sampled at 25 frames/s.

### Data analysis

All data analyses were performed in Python (Python Software Foundation, https://python.org/, version 3.8). A second order Butterworth lowpass filter at 12 Hz was used for the pelvis and trunk and a lowpass filter at 50 Hz was used for the upper arm. The Euclidean norm of the triaxial filtered gyroscope angular velocity was calculated to assess the peak angular velocity (ω) per segment in degrees per second. The time interval (milliseconds) between the peaks of (1) the pelvis and trunk angular velocities and (2) the trunk and upper arm angular velocities were calculated according to the definition that positive values indicated that the peak angular velocity of a more distally located segment occurred after the peak angular velocity of the preceding adjacent segment (Equation 1).

(1) $$\mathrm{Tim}e_{\mathrm{proximal},\mathrm{distal}}\;=\;\mathrm{Tim}e_{\mathrm{distal}}\text{–}\;\mathrm{Tim}e_{\mathrm{proximal}}$$

Using the PlaySight system, the 2D coordinates of the ball bounce positions were defined with axes aligned with the centre service line and the singles side line. These coordinates were subsequently used to categorize the accuracy based on bounces in four pre-defined target areas within the service box. These four areas were predefined in line with the expert opinions of coaches and technical staff when considering optimal and less optimal ball bounce areas for tennis serves. The four areas were defined differently for serves aimed at T, body or wide, and for first and second serves. Depending on the target area, the highest possible score was nine points, followed by six, three, one, and zero points when the ball did not bounce in the correct service box (Appendix, Figure A1).

### Statistical analysis

Significant differences between the first and de second serve were tested with two-tailed paired samples *t*-tests. The 24 first and 24 s serves were averaged for each participant. The mean values of each participant were used in the statistical analysis. The standard deviation was also calculated and represent the between subject variances. The data were normally distributed according to the Shapiro-Wilks tests and visual inspections of the q-q plots. The confidence intervals were set at 95%. The data were analysed using the SciPy package in Python (16).

The association between participant's kinematics variables and serve performance variables per stroke was investigated using linear regression analysis with Generalized Estimating Equations (GEE) and an exchangeable working correlation structure. GEE analyses were applied to account for the dependency of the repeated serve strokes within participants. The residuals were checked for normality and heteroscedasticity. In separate regression analyses, magnitudes of peak angular velocities of the pelvis, trunk, and upper arm were the independent (predictor) variables, as well as the intersegmental timing between peak segment angular velocities of the pelvis-trunk, and trunk-upper arm per stroke. Outcome variables included the ball speed and accuracy. First and second serves were analysed separately. Regression coefficients and corresponding 95% confidence intervals (CI) were then determined using Wald chi-square tests. Alpha was set on 0.05 to assess significance. The data were analysed using the StatsModel package in Python (17).

## Results

All players performed at least 48 and a maximum of 51 strokes due to lets resulting in a total of 390 performed serves. Due to 43 unregistered serves by the PlaySight system a total of 347 serves were included in the analyses. Descriptive values of the assessed variables are shown in Table 1. The descriptives show that the magnitude of the peak angular velocities is increasing in the order of pelvis, trunk and upper arm. The intersegmental timing was negative for the pelvis-trunk peak angular velocities, but positive for the trunk-upper arm.

**Table 1:** Mean (SD) of the serve performance indicators, peak angular velocities (deg/s) and the intersegmental timing (ms) for the total, first and second serve.

	First serve		Second serves		*t*-statistic	*p*-value
Serve performance						
Ball speed (kph)	175.1	(12.3)	145.7	(12.9)	13	<0.001
Percentage in/out (%)	55.3	(8.9)	79.5	(15.5)	−5.6	<0.001
Accuracy (points)	2.8	(0.6)	4.4	(1.1)	−4	<0.01
Peak angular velocities (deg/s)						
Pelvis	586.8	(58.4)	541.8	(49.4)	6.6	<0.001
Trunk	897.2	(152.9)	846.2	(146.3)	3.7	<0.01
Upper arm	3,206.6	(807.8)	2,719.8	(715.1)	6.9	<0.001
Intersegmental timing (ms)						
Time _Pelvis, Trunk_	−28.3	(33.5)	−28.9	(27.0)	0.1	0.9
Time _Trunk, Upper Arm_	124.5	(14.3)	127.3	(13.4)	−1.8	0.1

The last two columns show the resulting t-statistics and the *p*-values of the paired-sampled *t*-tests.

### First serve vs. second serve

Table 1 shows the paired sample *t*-test results of the first serve compared with the second serve. The first serve shows significantly higher ball speeds, but lower serve percentages and accuracy values (target area points) compared to the second serve.

**Figure 2:**
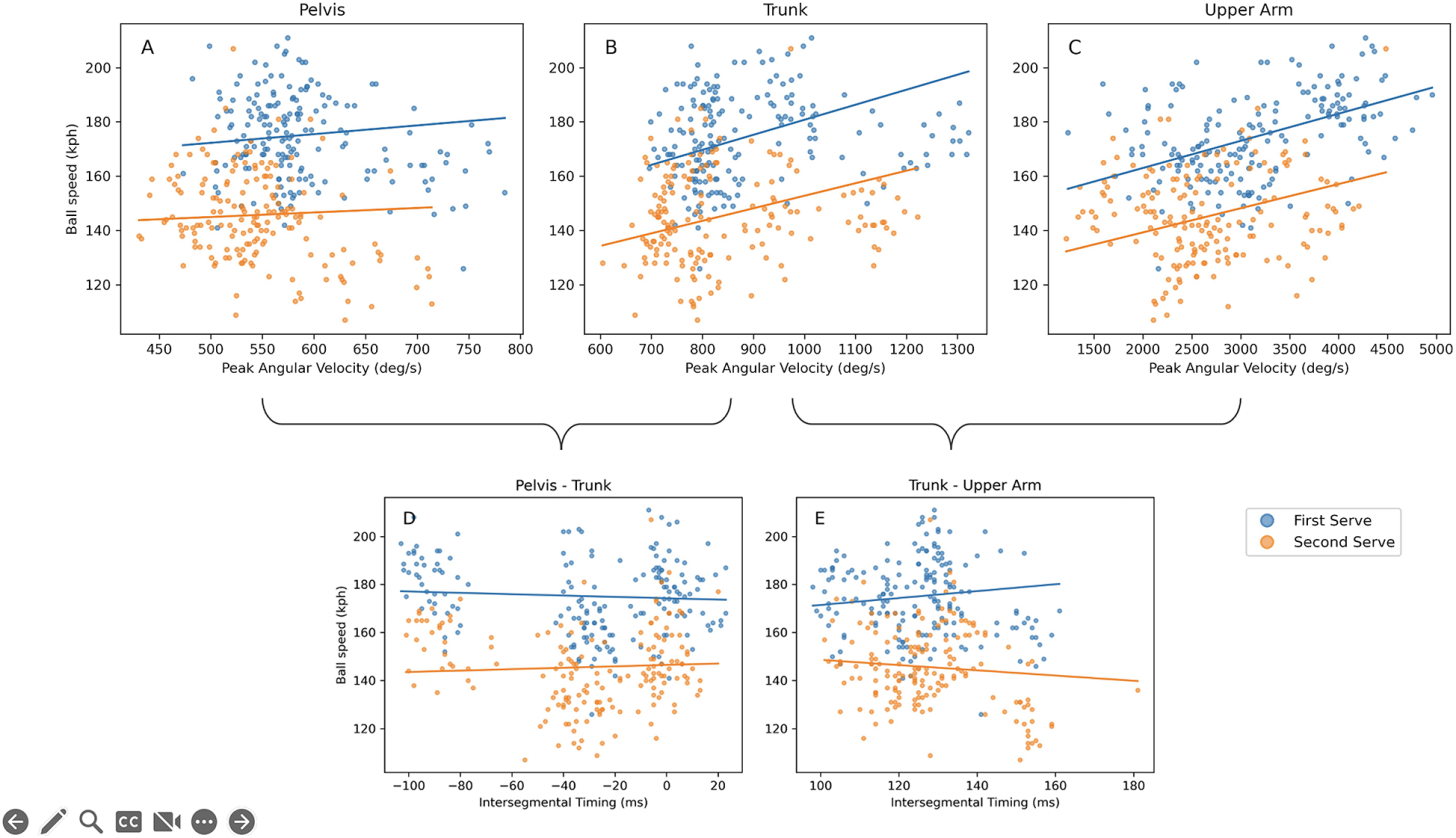
Scatter plots of ball speed in association with the predictor variables. Each dot is a single tennis serve. The blue dots represent the first serves, and the orange dots the second serves. Blue lines resemble the estimated regression lines for first serves, orange lines for the second serves. **(A–C)** show the peak angular velocities of the pelvis, trunk and upper arm, respectively. The intersegmental timing of the pelvis-trunk is shown in **(D)** and trunk-upper arm in **(E)**.

Peak angular velocities were significantly higher for the first serve (Figure 2). The intersegmental timing did not show significant differences between the first and the second serve.

### Serve performance in relation with (inter)segmental rotations and timing

Observed values and estimated regression lines from the GEE regression models of the associations between magnitudes of peak angular velocity per segment and intersegmental timing with serve performance variables (ball speed and accuracy) are shown in Figure 2.

### Ball speed

Table 2 shows regression coefficients and the corresponding 95% CI and significance levels for the predictor variables in relation to ball speed. A significant positive association was observed between ball speed and trunk and upper arm peak angular velocities in both first and second serves (Figures 1B,C). The value of the coefficient b_1_ (0.055) of the trunk angular velocity for the first serve shows that professional tennis players who show a 10/0.055, or ∼181 deg/s higher trunk angular velocity for the first serve and 271 deg/s for the second serve, serve 10 kph faster. A 1,000 and 1,111 deg/s higher value of upper arm angular velocity, for the first and second serve respectively, is associated with a 10 kph faster ball speed.

**Table 2:** Ball speed (kph) in association with magnitude of peak angular segmental velocities(deg/s) and intersegmental timing (ms), based on simple GEE regression analysis (ball speed (kph) = b_0_ + b_1_ * predictor (deg/s or ms)) (n_first serves_ = 177, n_second serves_ = 170).

Predictor	Serve	b_0_	b_1_	95% CI b_1_	*P*-value
Peak angular velocities (deg/s) of					
Pelvis	First	156.2	0.032	[−0.048, 0.112]	0.43
	Second	136.6	0.017	[−0.025, 0.058]	0.44
Trunk	First	125.4	0.055	[0.005, 0.106]	<0.05
	Second	106.6	0.046	[0.002, 0.090]	<0.05
Upper arm	First	143.0	0.010	[0.003, 0.017]	<0.01
	Second	121.5	0.009	[0.002, 0.016]	<0.01
Intersegmental timing (ms) between					
Pelvis-trunk	First	174.3	−0.028	[−0.079, 0.023]	0.28
	Second	146.5	0.030	[−0.009, 0.069]	0.14
Trunk - upper arm	First	157.1	0.143	[−0.234, 0.521]	0.45
	Second	159.5	−0.109	[−0.378, 0.160]	0.43

No significant associations were observed between ball speed and the intersegmental timing variables (Figures 2F,G).

### Accuracy

Table 3 shows regression coefficients and the corresponding 95% CIs and significance levels for the predictor variables in relation to accuracy. Trunk peak angular velocity was positively significantly associated (b1 = 0.002) with accuracy in the first serve, whereas it was negatively significantly associated (b1 = −0.002) in the second serve. This indicates that a 500 deg/s higher value in trunk peak angular velocity for the first and a 500 deg/s lower value for the second serve is associated with a 1-point higher accuracy score.

**Table 3:** Accuracy in association with magnitude of segmental peak angular velocities (deg/s) and intersegmental timing (ms), based on simple regression analysis (accuracy = b_0_ + b_1_ * predictor) with GEE (n_first serves_ = 177, n_second serves_ = 170).

Predictor	Serve	b_0_	b_1_	95% CI of b_1_	*P*-value
Peak angular velocities					
Pelvis	First	3.31	0.003	[−0.003, 0.009]	0.37
	Second	4.43	0.002	[−0.004, 0.008]	0.46
Trunk	First	3.30	0.002	[0.000, 0.003]	<0.01
	Second	7.07	−0.002	[−0.003, −0.000]	<0.05
Upper arm	First	6.24	0.000	[−0.001, 0.000]	0.19
	Second	6.55	0.000	[−0.001, 0.000]	0.24
Intersegmental timing					
Pelvis-trunk	First	5.50	0.014	[0.006, 0.022]	<0.001
	Second	5.31	−0.009	[−0.025, 0.007]	0.27
Trunk - upper arm	First	4.12	0.007	[−0.006, 0.020]	0.30
	Second	7.53	−0.015	[−0.037, 0.007]	0.18

The pelvis-trunk intersegmental timing was significantly associated (b1 = 0.014) with accuracy for the first serve. This regression coefficient shows that a 72 ms longer intersegmental timing is associated with a 1-point higher accuracy score (Figure 2).

## Discussion

The first aim of this study was to investigate if the upper body segments' peak angular velocities and their intersegmental timing were different between first and second tennis serves. The results showed that the peak angular velocities of the upper body segments were significantly higher for the first compared to the second serve. No differences were found in the intersegmental timing between the two serves. The second aim was to investigate if the tennis serve performance was associated with segmental peak angular velocities and the intersegmental timing between these peak angular velocities. Ball speed was associated with the trunk and upper arm rotations. Intersegmental timings were not associated with ball speed. Accuracy was associated with the trunk peak angular velocity. Pelvis-trunk intersegmental timing was associated with accuracy in the first serve.

### First and second serve

The current findings underline the difference in performance and kinematics between the first and second serve in professional tennis players. Higher ball speeds and lower serve percentage were found in the first serve (13), which is in line with a tennis match. Most tennis players hit their first serve flat and their second serves with more ball rotation (“kick” or “slice”) (18). In addition, the racket position at ball impact is different for serve types (19). Our results might explain that hitting a kick or slice serve changes segmental angular velocities early in the kinetic chain to position the racket at another position than the flat serve, without changing the intersegmental timing.

### Segmental peak angular velocities

The magnitudes of the peak angular velocity of the pelvis, trunk and upper arm increased in a proximal-to-distal order. The pelvis' peak angular velocity was not related to ball speed. Interestingly, it was significantly different between the first and second serve. It seems that the pelvis is a stable base for the trunk and the upper arm to rotate on. Higher trunk and upper arm peak angular velocities are associated with higher ball speeds in professional tennis players. This agrees with previous studies that underlined the role of trunk and upper limb angular velocities with achieving high ball speed in the tennis serve (20–22). To achieve an increase in ball speed of 1kph, an increase of 18 deg/s for the trunk and 100 deg/s for the upper arm is required in the first serve. These numbers are based on a group level of professional tennis players; some individuals might benefit more than other players. Trunk peak angular velocity is associated with accuracy, players with a higher trunk peak angular velocity were more accurate on the first serve. Instead in the second serve is an increase in trunk peak angular velocity associated with less accuracy. These findings suggest that, in a professional tennis player's serve performance, a more proximal body segment (such as the trunk) may have a relatively higher impact on the serve's accuracy compared to a more distal segment (upper arm), which plays a greater role in ball speed. In contrast, Whiteside et al. (23) showed that body kinematics were not different between a fault (net serve) and a good serve, but the projection angle of the ball appears closely related to serve outcome (23). We investigated the accuracy of serves that were only hit in the correct serve box, which might explain this difference in observed results. The trunk seems an important segments in relation to tennis serve performance.

### Intersegmental timing

The intersegmental timing was not associated with ball speed. Remarkably, and in contrast to studies that identified a proximal-to-distal segment sequence according to the summation of speed principle in the tennis serve (7, 8, 21, 24), we did not observe this sequence at group level. On average, the timing of the peak angular velocity of the pelvis occurred later in time than the trunk's (Figure 2F). This seemingly out of sequence motion of the pelvis and trunk was also observed in professional players in a study by Fleisig et al. (2). The upper arm followed the trunk in the proximal-to-distal sequence; however, the intersegmental timing was not related to ball speed. Marshall and Elliot (25) mentioned that the upper arm's maximal internal rotation occurs just before ball impact and is not following the proximal to distal sequence and thus the summation of speed principle (25). Alternatively, the kinetic chain can be described by the principle of optimal coordination of partial momenta (5). This principle states that all segments must reach the same peak angular velocity at the same time. Describing the tennis serve to this principle seems also not possible as our results showed that the segment angular velocities are not rotating with the same angular speed at the distal end (Table 1). Instead of explaining the tennis serve by a kinetic chain that is following a single proximal-to-distal sequence as stated in previous literature (6) or partia momenta. We suggest subdivided the tennis serve into different parts. While the pelvis-trunk and upper arm–forearm might follow the principle of partial momenta and the trunk-upper arm likely move in line with the summation of speed principle. In other overhead sports, like baseball pitching, the intersegmental timing is related to ball speed (9, 10). The reason why in the present study no such relationship in the tennis serve was observed might be explained by the fact that the tennis serve is hitting a ball and not releasing the ball from the hand as in baseball pitching. Tennis players can predict the endpoint for ball impact, as the ball is tossed in the air and follows a parabolic curve. The position of hitting the ball is essential for the serve outcome (23), this will fix the endpoint of the distal segment. This fixed endpoint might explain that the intersegmental timing is also fixed. To investigate this hypothesis, future studies could investigate if the intersegmental timing is constant during the serve while instructed to hit the ball over a range of different speeds and aiming at the same target.

Kinematics were assessed with the use of IMUs, rather than a more commonly used 3D marker-based motion capture system. IMUs measures directly the angular velocities. By using IMUs on the court, the data is collected fast and easy on the tennis court in real tennis situations. The collected data can be transferred to an app and subsequently provide coaches and players with information on the court (26). In addition, coaches who are aiming to improve the serve performance by adjusting the biomechanics of their athletes, can use the IMU sensor system to monitor if the training and their feedback was successful. Because, with the IMU sensor system they can easily quantify if athletes reached higher peak angular velocities in the trunk and upper arm, something trainers cannot see with their eyes.

To enhance ecological validity, the participants performed the test on the court and were instructed to hit a ball just like in a tennis match. However, a limitation is that we could not confirm whether they perform like in a tennis match. In baseball research one study showed that the highest league players threw slower compared to a lower league, likely due to a lack of motivation (27). Implementing a measurement setup during an actual match could further increase ecological validity. The use of wearable sensors presents a promising opportunity for future research.

This study included eight professional tennis players; this relatively small sample size is a limitation. Logically, there are fewer professional players, which makes it difficult to include more in a study. However, we were able to perform a cross-sectional design instead of just a case study. In addition, by collecting and analysing data of each serve, we could perform a GEE statistical test that included all serves. Instead of having 8 data points per outcome variable, 347 data points were included. This study included 2 female and 6 male players. In general, the tennis serve ball speed is lower in female players. For the external validity it would have been better to have an equal number of female and male participants. However, in our study the GEE takes individual differences into account. Furthermore, we only investigated adult professional players, so it is uncertain whether the results on intersegmental timing can be extrapolated to other skill levels and younger players. It is known that beginners show different kinematics (28). Future research should investigate if trunk and upper limb rotations, as well as their intersegmental timing, differ between female and male, levels of play and age groups during the tennis serve. Due to the newly developed wearable setup and analysis this study, it is now easier to answer these questions.

## Conclusion

This study highlights the role of the kinetic chain in tennis serve performance in professional players, based on directly measured kinematics with IMUs on the court. The tennis serve body motion is not entirely performed in line with the proximal-to-distal sequence. This seems confirmed by the fact that intersegmental timings for the first and second serve are similar and shows no association with ball speed. It is generally known, and confirmed by our results, that upper arm rotation is important to produce high ball speeds in the tennis serve. In addition, the trunk rotation, proximal with respect to the upper arm in the kinetic chain showed associations with ball speed. In contrast to the arm, we uncovered that the trunk rotation exhibited an association with the accuracy. Future research should uncover further the role of the trunk in the tennis serve. With the use of IMU systems and the developed methodology it is possible to measure and quantify trunk and arm movements on the court.

## Data Availability

The raw data supporting the conclusions of this article will be made available by the authors, without undue reservation.

## References

[1] Mecheri S Rioult F Mantel B Kauffmann F Benguigui N. The serve impact in tennis: first large-scale study of big hawk-eye data. Stat Anal Data Min. (2016) 9:310–25. 10.1002/sam.11316

[2] Fleisig G Nicholls R Elliott B Escamilla R. Tennis: kinematics used by world class tennis players to produce high-velocity serves. Sports Biomech. (2003) 2:51–64. 10.1080/14763140308522807 14658245

[3] Kovacs M Ellenbecker T. An 8-stage model for evaluating the tennis serve: implications for performance enhancement and injury prevention. Sports Health. (2011) 3:504–13. 10.1177/1941738111414175 23016050 PMC3445225

[4] Elliott B Fleisig G Nicholls R Escamilia R. Technique effects on upper limb loading in the tennis serve. J Sci Med Sport. (2003) 6:76–87. 10.1016/S1440-2440(03)80011-7 12801213

[5] Putnam CA. Sequential motions of body segments in striking and throwing skills: descriptions and explanations. J Biomech. (1993) 26:125–35. 10.1016/0021-9290(93)90084-R 8505347

[6] Eygendaal D Rahussen FTG Diercks RL. Biomechanics of the elbow joint in tennis players and relation to pathology. Br J Sports Med. (2007) 41:820–3. 10.1136/bjsm.2007.038307 17638843 PMC2465285

[7] Elliott B Marsh T Blanksby B. A three-dimensional cinematographic analysis of the tennis serve. J Appl Biomech. (1986) 2:260–71. 10.1123/ijsb.2.4.260

[8] Ben Kibler W. Biomechanical analysis of the shoulder during tennis activities. Clin Sports Med. (1995) 14:79–85. 10.1016/S0278-5919(20)30259-3 7712559

[9] Urbin MA Fleisig GS Abebe A Andrews JR. Associations between timing in the baseball pitch and shoulder kinetics, elbow kinetics, and ball speed. Am J Sports Med. (2013) 41:336–42. 10.1177/0363546512467952 23204507

[10] van der Graaff E Hoozemans MMJM Nijhoff M Davidson M Hoezen M Veeger DHEJ. Timing of peak pelvis and thorax rotation velocity in baseball pitching. J Phys Fit Sports Med. (2018) 7:269–77. 10.7600/jpfsm.7.269

[11] Martin C Bideau B Bideau N Nicolas G Delamarche P Kulpa R. Energy flow analysis during the tennis serve: comparison between injured and noninjured tennis players. Am J Sports Med. (2014) 42(11):2751–60. 10.1177/0363546514547173 25167995

[12] Gomaz L Veeger D van der Graaff E van Trigt B van der Meulen F. Individualised ball speed prediction in baseball pitching based on imu data. Sensors. (2021) 21:7442. 10.3390/s21227442 34833517 PMC8622425

[13] Chow J Carlton L Lim Y-T Chae W-S Shim J-H Kuenster ANN Comparing the pre-and post-impact ball and racquet kinematics of elite tennis players’ first and second serves: a preliminary study. J Sports Sci. (2003) 21:529–37. 10.1080/0264041031000101908 12848387

[14] Lyons M Al-Nakeeb Y Hankey J Nevill A. The effect of moderate and high-intensity fatigue on groundstroke accuracy in expert and non-expert tennis players. J Sports Sci Med. (2013) 12:298. 24149809 PMC3761827

[15] Whiteside D Reid M. Spatial characteristics of professional tennis serves with implications for serving aces: a machine learning approach. J Sports Sci. (2017) 35:648–54. 10.1080/02640414.2016.1183805 27189847

[16] Gommers R Virtanen P Burovski E Weckesser W Oliphant TE Haberland M scipy/scipy: SciPy 1.9. 0. Zenodo. (2022).

[17] Seabold S Perktold J. Statsmodels: econometric and statistical modeling with python. Proceedings of the 9th Python in Science Conference; Austin, TX (2010). 10–25080.

[18] Sakurai S Reid M Elliott B. Ball spin in the tennis serve: spin rate and axis of rotation. Sports Biomech. (2012) 12(1):23–9. 10.1080/14763141.2012.671355 23724605

[19] Sheets AL Abrams GD Corazza S Safran MR Andriacchi TP. Kinematics differences between the flat, kick, and slice serves measured using a markerless motion capture method. Ann Biomed Eng. (2011) 39:3011–20. 10.1007/s10439-011-0418-y 21984513

[20] Elliott BC Marshall RN Noffal GJ. Contributions of upper limb segment rotations during the power serve in tennis. J Appl Biomech. (1995) 11:433–42. 10.1123/jab.11.4.433

[21] Bahamonde RE. Changes in angular momentum during the tennis serve. J Sports Sci. (2000) 18:579–92. 10.1080/02640410050082297 10972409

[22] Martin C Kulpa R Delamarche P Bideau B. Professional tennis players’ serve: correlation between segmental angular momentums and ball velocity. Sports Biomech. (2013) 12:2–14. 10.1080/14763141.2012.734321 23724603

[23] Whiteside D Elliott B Lay B Reid M. A kinematic comparison of successful and unsuccessful tennis serves across the elite development pathway. Hum Mov Sci. (2013) 32:822–35. 10.1016/j.humov.2013.06.003 23973088

[24] Wagner H Pfusterschmied J Tilp M Landlinger J von Duvillard SP Müller E. Upper-body kinematics in team-handball throw, tennis serve, and volleyball spike. Scand J Med Sci Sports. (2014) 24:345–54. 10.1111/j.1600-0838.2012.01503.x 22813080

[25] Marshall RN Elliott BC. Long-axis rotation: the missing link in proximal-todistal segmental sequencing. J Sports Sci. (2000) 18:247–54. 10.1080/026404100364983 10824641

[26] van Trigt B. Keep the Pitcher’s Elbow Load in the Game: Biomechanical Analysis of Injury Mechanisms in Baseball Pitching Towards Injury Prevention. De Bilt: Global Academic Press (2023).

[27] Fleisig GS Chu Y Weber A Andrews JR. Variability in baseball pitching biomechanics among various levels of competition. Sports Biomech. (2009) 8:10–21. 10.1080/14763140802629958 19391491

[28] Hernández-Davó JL Moreno FJ Sanz-Rivas D Hernández-Davó H Coves Á Caballero C. Variations in kinematic variables and performance in the tennis serve according to age and skill level. Int J Perform Anal Sport. (2019) 19(5):749–62. 10.1080/24748668.2019.1653036

