# Supplementary material for: Uncovering the hidden mechanics of upper body rotations in tennis serves using wearable sensors on Dutch professional players
Source: Front Sports Act Living. 2025 Jan 7;6:1463299. doi: 10.3389/fspor.2024.1463299 (PMC11746891; doi:10.3389/fspor.2024.1463299)

# Supplementary materials

**Target areas STT
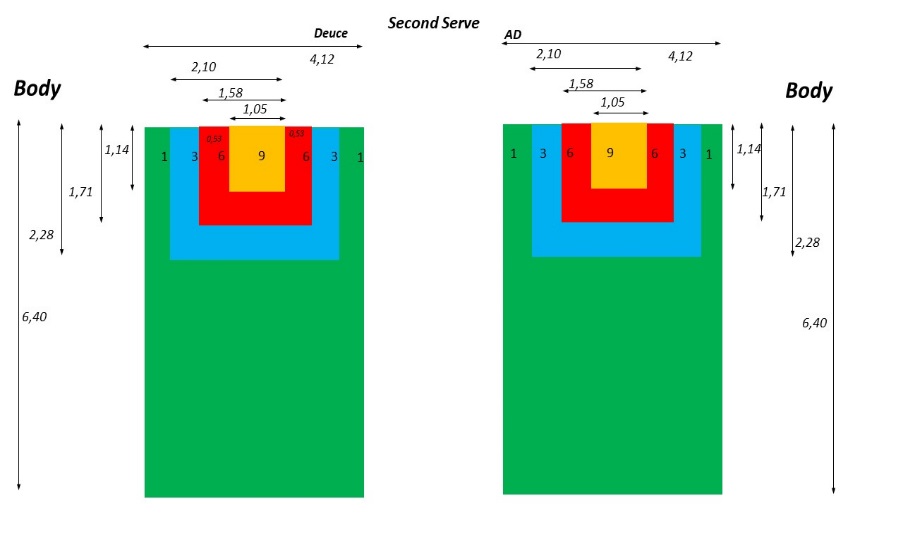
**
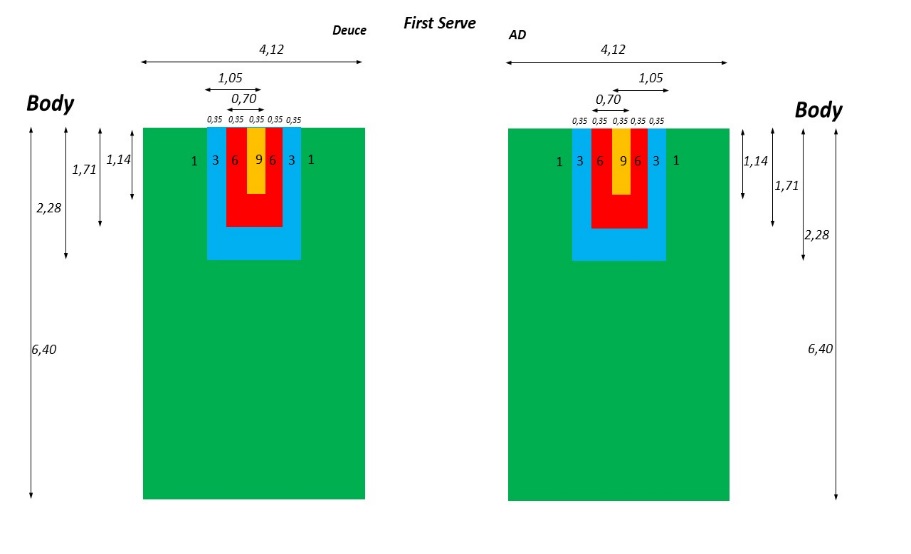


**
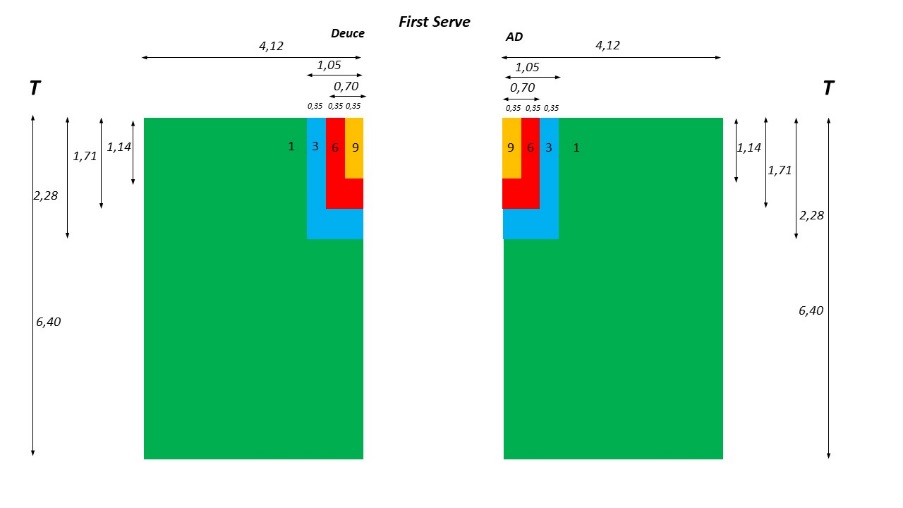

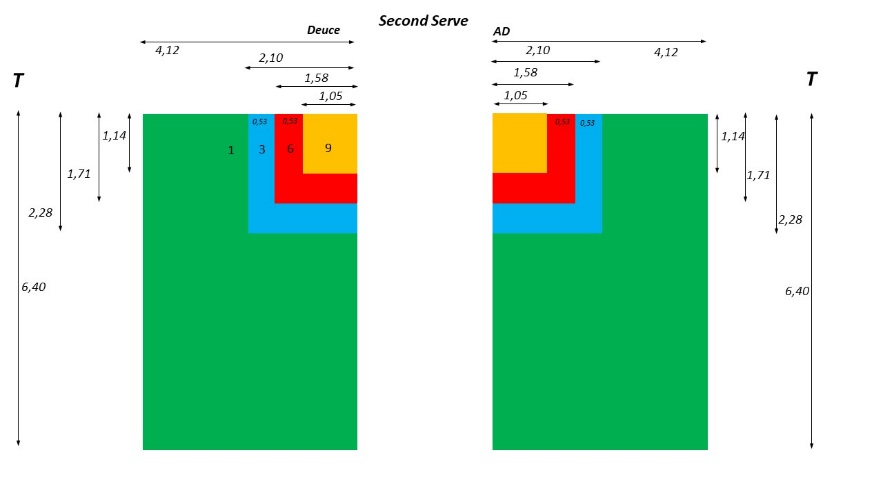

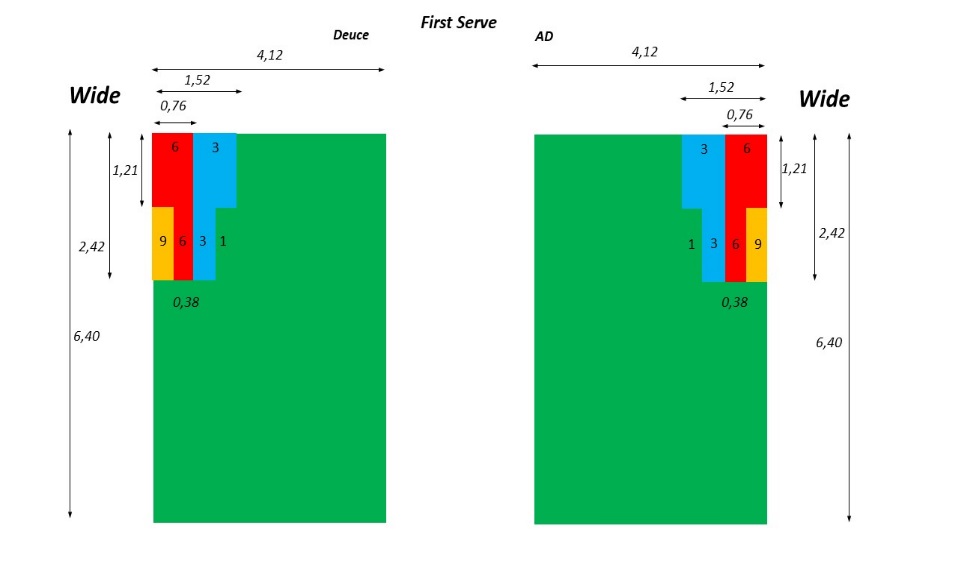

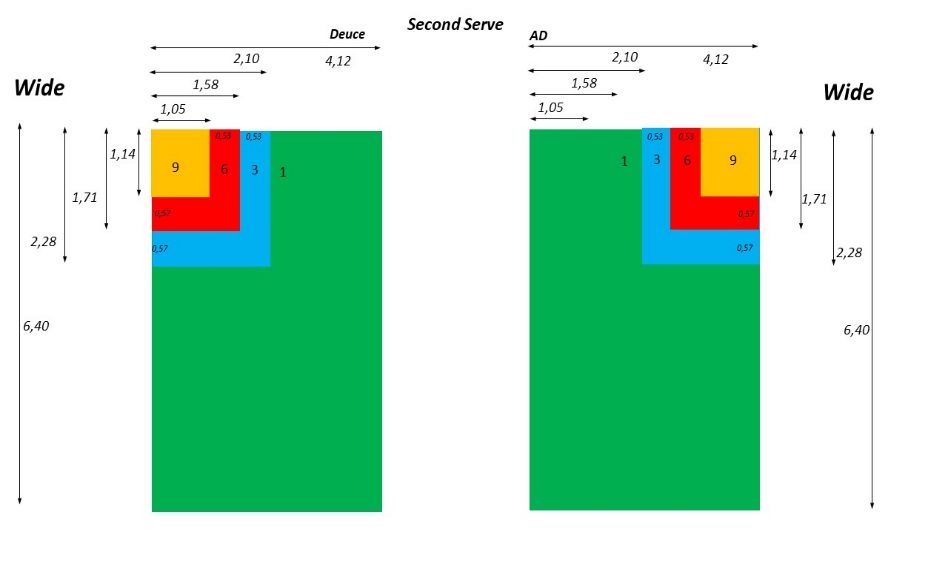
**

**Figure A1.** Target areas, for first (left column) and second serves (right column) for the deuce and Advantage (AD) side. The first, second and third row are the target areas for the wide, body and T, respectively. The numbers are in meters.

**Protocol STT**

**
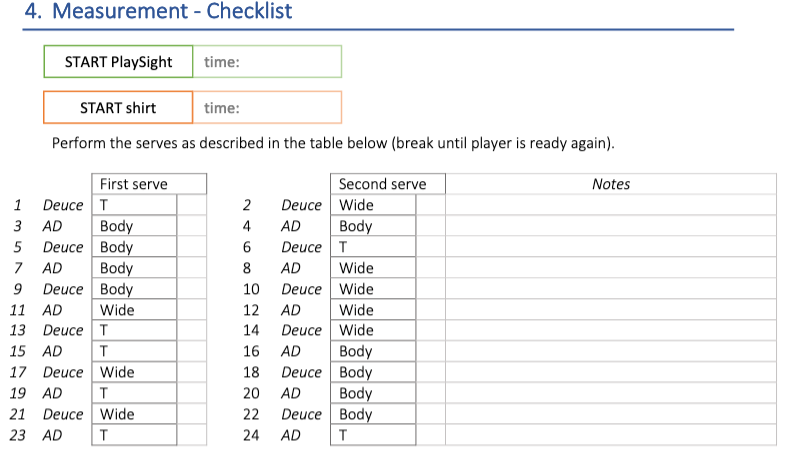
**


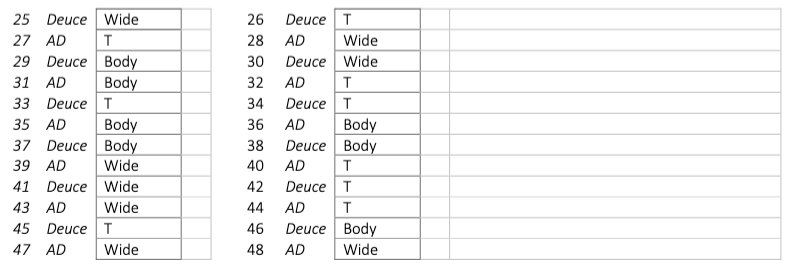

Supplement: Supplementary file 1 [file Datasheet1.docx]
